# Brainstem Toxicity in Pediatric Patients Treated with Protons Using a Single-vault Synchrocyclotron System

**DOI:** 10.14338/IJPT-22-00008.1

**Published:** 2022-06-03

**Authors:** Inema Orukari, Stephanie Perkins, Tianyu Zhao, Jiayi Huang, Douglas F. Caruthers, Sai Duriseti

**Affiliations:** 1 Washington University in St. Louis School of Medicine, St. Louis, Missouri, USA; 2 Department of Radiation Oncology, Washington University School of Medicine/Barnes Jewish Healthcare, St. Louis, Missouri, USA

**Keywords:** pediatrics, brainstem, synchrocyclotron, radionecrosis, passive-scatter

## Abstract

**Purpose:**

Cranial radiation therapy remains an integral component of curative treatment for pediatric patients with brain tumors. Proton beam radiation therapy (PBT) can limit collateral radiation dose to surrounding normal tissue, thus reducing off-target exposure while maintaining appropriate tumor coverage. While PBT offers significant advantages over photon therapy for pediatric patients with intracranial malignancies, cases of brainstem necrosis after PBT have raised concerns that PBT may pose an increased risk of necrosis over photon therapy. We investigated the incidence of brainstem necrosis at our institution in children treated with PBT for intracranial malignancies.

**Patients and Methods:**

Patients with pediatric brain tumor treated with passively scattered PBT, using a gantry-mounted, synchrocyclotron single-vault system between 2013 and 2018, were retrospectively reviewed. Inclusion criteria included patients 21 years of age or younger who received a minimum 0.1 cm^3^ maximum brainstem dose of 50 Gray relative biological effectiveness (GyRBE). Patients were assessed for “central nervous system necrosis” in the brainstem per the Common Terminology Criteria for Adverse Events (CTCAE), version 5.0 (US National Cancer Institute, Bethesda, Maryland) criteria.

**Results:**

Fifty-eight patients were included for analysis. The median age was 10.3 years. Twenty-one (36.2%) patients received craniospinal irradiation. Thirty-four (58.6%) patients received chemotherapy. The median prescription radiation dose was 54 GyRBE. Regarding published dosimetric constraints used at 3 separate proton centers, the goal brainstem D50% <52 GyRBE was exceeded in 23 (40%) patients, but the brainstem Dmax <58 GyRBE was not exceeded in any patients. No patient experienced grade ≥2 brainstem injury. One patient demonstrated radiographic changes consistent with grade 1 toxicity. This patient had myeloablative chemotherapy with tandem stem cell rescue before PBT.

**Conclusion:**

Our data demonstrates a low risk of any brainstem injury in children treated with passively scattered PBT using a single-vault synchrocyclotron.

## Introduction

Pediatric brain tumors are the most common solid tumors of childhood and comprise a wide variety of tumor types. With modern therapy, overall survival at 5 years is approximately 75% [1]. Radiation therapy remains an integral component of curative treatment; however, significant sequelae of radiation include neurocognitive deficits, neuroendocrine abnormalities, vasculopathy, hearing loss, permanent hair loss, and risk of secondary malignancies due to collateral exposure of nearby normal tissue [2–6]. Proton beam radiation therapy (PBT) can reduce radiation dose to the surrounding normal tissues while maintaining appropriate tumor coverage. This normal tissue sparing is achieved through the unique physical characteristics of protons that allow control over the dose deposition at a specified depth that is not achievable with photon therapy [7, 8].

PBT plans use the spread-out Bragg peak (SOBP) to achieve the desired dose distribution that conforms to the target volume [9]. The individual Bragg peak components of the SOBP tend to have the highest linear energy transfer (LET) at the distal end of the beam range, which translates to the SOBP having the highest LET at the end of its profile.

While PBT offers significant advantages over photon therapy for pediatric cranial radiation, cases of brainstem necrosis after PBT over the past 10 years have raised some concerns regarding the potential of a unique risk profile of necrosis that differed from the low risk of necrosis seen following photon therapy [10–13]. These data led to the formation of a National Cancer Institute (NCI) Workshop on Proton Therapy for Children that focused on the incidence of brainstem injury following PBT, and dosimetric parameters to prevent it. The article from this workshop details the analysis of the literature and reports a low risk of brainstem necrosis when brainstem dosimetric guidelines are instituted and followed [14, 15].

At our institution, we began treating pediatric brain tumors with PBT before the development of these brainstem constraints recommended in the NCI Workshop. Additionally, our proton therapy system is unique in that it uses a compact synchrocyclotron technology with unique beam characteristics. For these reasons, we investigated the incidence of Common Terminology Criteria for Adverse Events (CTCAE), version 5 (US National Cancer Institute, Bethesda, Maryland) brainstem toxicity at our institution in children treated with PBT for intracranial malignancies.

## Patients and Methods

With approval of the Washington University Institutional Review Board, patients with pediatric brain tumor treated with passively scattered PBT between 2013 and 2018 were retrospectively reviewed. Inclusion criteria included patients 21 years of age or younger who received a minimum 0.1 cm^3^ maximum brainstem dose of 50 Gray relative biological effectiveness (GyRBE). Patient characteristics, diagnosis, extent of surgery, and use of chemotherapy were gathered for all patients.

Brainstem dosimetric data were obtained from the treatment planning software, including Dmax, 0.1 cm^3^ maximum dose, dose to 10% of the brainstem (D10%), and brainstem D50%. All patients were followed up for >1 year after completion of therapy. Patients were assessed for “central nervous system necrosis” in the brainstem per CTCAE version 5.0 criteria (**Table 1**). Postradiation treatment change was defined as magnetic resonance imaging (MRI) changes in the absence of disease progression or symptoms. Radiation necrosis was defined per Indelicato et al [15] as follows: (1) new or progressive symptoms and/or signs after irradiation involving motor weakness or palsies of cranial nerves V to VII or IX to XII with (2) corresponding radiographic abnormalities within the brainstem and (3) absence of local disease progression. Our analysis included evaluation of the number of our patients who exceeded published dose constraints from 3 separate proton centers [14].

**Table 1.** CTCAE version 5.0: Central nervous system necrosis is defined as a “disorder characterized by a necrotic process occurring in the brain and/or spinal cord.”

**Grade**	**Criteria and intervention**
1	Asymptomatic; clinical or diagnostic observations only; intervention not indicated
2	Moderate symptoms; corticosteroids indicated
3	Severe symptoms; medical intervention indicated
4	Life-threatening consequences; urgent intervention indicated
5	Death

**Abbreviation:** CTCAE, Common Terminology Criteria for Adverse Events.

All patients received passive scatter PBT using a gantry-mounted synchrocyclotron single-vault system. All patients were treated with 3 to 4 fields and each field was treated daily. For patients receiving craniospinal irradiation (CSI), 2 equally weighted opposite lateral fields were used for the cranial fields and 1 or 2 posterior fields for the spinal fields. Posterior fossa boosts following CSI were always a 3-field plan with posterior-anterior, right posterior oblique, and left posterior oblique fields.

## Results

Patient demographic data are listed in **Table 2**. Sixty-three patients were eligible for inclusion. Four of these patients died before 1-year follow-up and 1 patient was lost to follow-up and they were thus excluded from analysis. Of note, the 4 patients who died before the 1-year follow-up did not die from brainstem radionecrosis, nor did they demonstrate brainstem changes indicative of radiation toxicity. The remaining 58 patients had a median age of 10.3 years (range, 1-21.9 years). Craniospinal irradiation was delivered to 21 (36.2%) patients, and 15 (71.4%) of these patients received 23.4 GyRBE, whereas 6 (28.6%) of these patients received 36 GyRBE. Chemotherapy was delivered to 34 patients (58.6%); of these, 2 patients (5.9%) received myeloablative chemotherapy with stem cell rescue before radiation.

**Table 2.** Patient demographics and clinical factors.

**Factor**	**Value**
Total eligible patients, n (%)	58 (100)
Age, median (range), y	10.3 (1–21.9)
Sex, n	
Male	38
Female	20
Histology, n (%)	
Medulloblastoma	17 (29)
Craniopharyngioma	10 (17)
Ependymoma	9 (16)
Juvenile pilocytic astrocytoma	6 (10)
Optic pathway glioma	2 (3)
Germ cell tumor	2 (3)
WHO II oligodendroglioma	2 (3)
Other	9 (16)
Extent of surgery, n (%)	
Gross total/near total resection	52 (90)
Subtotal resection	2 (3)
Biopsy-only	4 (7)
Radiation dose, median (range), GyRBE	54 (50.4–60)
Received CSI, n (%)	21 (36)
23.4 GyRBE CSI	15 (71)
36 GyRBE CSI	6 (29)
Chemotherapy, n (%)	
Pre-PBT chemotherapy	13 (22)
Concurrent chemotherapy	19 (33)
Adjuvant chemotherapy	20 (34)
Myeloablative chemotherapy with stem cell rescue	2 (3)
Intrathecal methotrexate	1 (2)

**Abbreviations:** WHO, World Health Organization; CSI, craniospinal irradiation; PBT, proton beam radiation therapy.

The median prescription radiation dose was 54 GyRBE (range, 50.4-60 GyRBE). Mean brainstem dose was 35.5 GyRBE (range, 3.3-55.2 GyRBE). Average Dmax to the brainstem was 54.5 GyRBE (range, 51.1-57.1 GyRBE). Average D10% to brainstem was 50.4 GyRBE (range, 15.5-55.7 GyRBE). Using dosimetric criteria from published constraints used at 3 separate proton centers (**Table 3**), the goal brainstem D50% <52 GyRBE was exceeded in 23 (40%) patients, but the brainstem Dmax <58 GyRBE was not exceeded in any patients.

**Table 3.** Dosimetric objectives to the brainstem from 3 separate proton centers.

**Institution**	**Dosimetric objective**	**Patients in this cohort who exceeded dosimetric constraint, n (%)**
Massachusetts General Hospital	Dmax < 58 GyRBE	0
	D50% < 52.4 GyRBE	23 (40)
University of Florida	0.1 cm^3^	
	< 56.6 GyRBE (goal)	5 (9)
	< 58 GyRBE (max)	0
	D10%	
	< 55.4 GyRBE (goal)	8 (14)
	< 56 GyRBE (max)	1 (2)
	D50%	
	< 52.4 GyRBE (goal)	23 (40)
	< 54 GyRBE (max)	9 (16)
MD Anderson	D50% < 52 GyRBE (goal)	23 (40)
	0.1 cm^3^ < 58 GyRBE (max)	0

No patient experienced CTCAE version 5.0, grade ≥2 brainstem injury. One (1.7%) patient had radiographic changes consistent with CTCAE version 5.0, grade 1 toxicity. This patient was diagnosed with atypical teratoid rhabdoid tumor before 1 year of age. Treatment included posterior fossa tumor resection followed by myeloablative chemotherapy with tandem stem cell rescue. PBT followed transplant and consisted of conformal posterior fossa radiation to 50.4 GyRBE to the surgical bed. The patient exceeded no goal or maximum constraints listed in **Table 3**. The radiographic findings are included in the **Figure**. Clinically, the patient was asymptomatic, and no treatment was given. MRI scans were repeated at 8-week intervals for 2 scans and the patient was examined in clinic for any signs of neurologic symptoms. The MRI findings resolved within 6 months without intervention.

**Figure.**
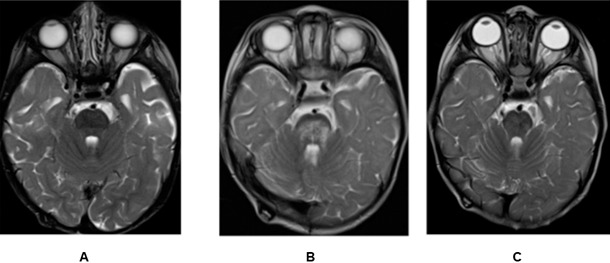
T2-FLAIR MRI images of single patient with CTCAE grade 1 brainstem changes (A) before radiation, (B) 7.5 weeks after radiation (scan demonstrating new onset patchy edema with microhemorrhages in the pons), and (C) 6 months following completion of radiation (MRI demonstrates resolution of brainstem findings). Abbreviations: CTCAE, Common Terminology Criteria for Adverse Events; FLAIR, fluid-attenuated inversion recovery; MRI, magnetic resonance imaging.

## Discussion

Our data indicate a low risk of any CTCAE version 5.0 brainstem injury in a cohort of children treated with passive scatter PBT using a synchrocyclotron. It is important to note that patients treated earlier in this cohort exceeded more recently published brainstem dose constraints. Despite this, there were no cases of symptomatic brainstem injury. Only 1 patient had radiographic changes after PBT, and this patient received myeloablative chemotherapy before PBT. Given the unique attributes of synchrocyclotron technology, these data are important in demonstrating no evidence of increased or unexpected toxicity to the brainstem.

Early publications of high-dose posterior fossa radiation in children indicated frequent postradiation MRI changes [11] with rare cases of severe/fatal radiation necrosis [15]. In 2018, following increasing concerns of reported cases of fatal radiation necrosis, an NCI Workshop meeting was held to discuss the current clinical data and experience with brainstem injury in PBT patients [14]. An analysis of 671 pediatric patients with posterior fossa tumor treated with PBT between 2006 and 2016 demonstrated a 1.3% risk of grade 3+ brainstem injury with 0.4% fatal brainstem necrosis. Further analysis by Indelicato et al [15] identified age <5 years and various dosimetric parameters (ie, brainstem Dmax >56.6 GyRBE) as predictive of symptomatic necrosis.

During the NCI Workshop, there was consensus that “differences in linear energy transfer (LET) (and, by implication, relative biological effectiveness [RBE]) when treating posterior fossa tumors anatomically adjacent to the brainstem contribute to brainstem injury.” Although the risk of brainstem necrosis is low for patients in whom Dmax constraints are respected, future therapy may rely on LET optimization to further reduce the risk for young patients requiring high volume or doses to the brainstem [16].

Passive scatter PBT relies on metal apertures to shape the proton beam as it enters the patient, and tissue compensators to adjust proton range, thus effecting an SOBP [17]. There are concerns regarding the higher RBE in an SOBP obtained with a passive scattering PBT plan, with rates of brainstem necrosis seen at higher rates in patients treated with passive scatter PBT. While the absolute rates of brainstem toxicity are low, prior groups have found a non-zero rate of symptomatic brainstem injury [15]. This is in contrast to our findings where only 1 patient experienced grade 1 brainstem injury, and there were no cases of symptomatic injury.

One possibility for the lack of any symptomatic brainstem injury is our specific method for delivery of proton therapy. These data are the first to report the risk of brainstem necrosis in pediatric patients treated with a single-vault synchrocyclotron system. A single-vault synchrocyclotron system allows production of high-energy protons at the expense of a wider energy spectrum exiting the snout due to unique energy modulation of the particles in the beamline [18]. Thus, while the doses delivered to our patients are the same as for patients treated with classic cyclotron or synchrotron systems, our beamline LET is lower at the end of range in a single-vault synchrocyclotron system [19]. In addition to the unique LET properties, beam and dose delivery characteristics are currently being studied by our group to evaluate LET and RBE in the brainstem as compared to other therapeutic proton delivery machines. Overall, it is important to note that the rate of any radiographic brainstem changes was low following high-dose posterior fossa PBT using synchrocyclotron technology.

This study is limited by the small number of patients in evaluating a rare complication. However, these data do include cohort patients at risk of necrosis, including infants, patients receiving myeloablative chemotherapy, and patients receiving radiation doses in excess of later published constraints. For the 1 case of postradiation MRI changes, the child was watched closely with MRI images every 8 weeks with physical examination. No intervention was required, and the imaging findings resolved spontaneously. Our current guidelines follow the University of Florida brainstem goal and acceptable constraints as detailed in the NCI Workshop article [14].
